# Experimental Investigation on Direct Micro Milling of Cemented Carbide

**DOI:** 10.3390/mi10020147

**Published:** 2019-02-22

**Authors:** Xian Wu, Liang Li, Ning He, Guolong Zhao, Jianyun Shen

**Affiliations:** 1 College of Mechanical Engineering and Automation, Huaqiao University, Xiamen 361021, China; jianyun@hqu.edu.cn; 2 College of Mechanical and Electrical Engineering, Nanjing University of Aeronautics and Astronautics, Nanjing 210016, China; liliang@nuaa.edu.cn (L.L.); drnhe@nuaa.edu.cn (N.H.); zhaogl@nuaa.edu.cn (G.Z.)

**Keywords:** micro milling, cemented carbide, surface quality, tool wear mechanism

## Abstract

Cemented carbide is currently used for various precise molds and wear resistant parts. However, the machining of cemented carbide still is a difficult challenge due to its superior mechanical properties. In this paper, an experimental study was conducted on direct micro milling of cemented carbide with a polycrystalline diamond (PCD) micro end mill. The cutting force characteristics, surface formation, and tool wear mechanisms were systematically investigated. Experimental results show that cemented carbide can be removed with ductile cutting utilizing the PCD tool with a large tool tip radius. Micro burrs, brittle pits, and cracks are the observed surface damage mechanisms. The tool wear process presents microchipping on the cutting edge and exfoliating on the rake face in the early stage, and then severe abrasive and adhesive wear on the bottom face in the following stage.

## 1. Introduction

Mold technology is an important method of mass production in various fields [1,2]. Cemented carbide offers many excellent features such as high hardness, high temperature resistance, and corrosion resistance. It is widely applied as mold materials and wear resistant parts. More and more molds are made of cemented carbide instead of steel to fulfill the demands of long mold life and high product quality [3,4,5]. Micromechanical cutting can produce miniature parts that have three-dimensional features such as narrow grooves, microcavities, and an aspheric surface [6,7,8,9]. After finishing, it can achieve the optical machining quality requirements for both geometric accuracy and surface roughness. Liu et al. [10] developed a prediction model and then studied the effects of cutting velocity and edge radius on the minimum uncut chip thickness in micro milling. Przestacki et al. [11] also proposed an approach to estimate the minimum uncut chip thickness during laser-assisted turning of tungsten carbide/nichrome (WC/NiCr) clad layers. Twardowski et al. [12] developed a surface roughness model considering dynamical cutter displacements in high-speed milling of hardened steel. Zhang and To [13] studied the effect of spindle vibration on surface generation based on the specialized model under the excitation of an intermittent cutting force during ultra-precision raster milling. Wojciechowski and Mrozek [14] investigated the dynamics of micro ball end milling with various tool inclination angles and optimized the machining parameters to obtain the minimization of cutting forces, vibrations, and surface roughness. 

However, compared with other materials, the superior mechanical properties of cemented carbide bring significant challenges such as excessive tool wear and poor surface quality during the microcutting process. However, with the development of ultra-hard tools, the direct cutting on cemented carbide becomes possible and attracts growing concern [15,16,17]. To obtain the machined surface of mirror quality, many previous studies have been focused on ductile cutting of cemented carbide. Liu et al. [18] reported that the ductile cutting surface and chip formation can be obtained when the uncut chip thickness is small enough during high-speed cutting of cemented carbide. Then, they [19] found the transition from ductile to brittle cutting as the cutting depth increased from zero to a critical value in the grooving of cemented carbide. Based on the energy balance criterion, Arif et al. [20] proposed an analytical model to predict the critical uncut chip thickness for a ductile-brittle transition in end milling of cemented carbide. Ultra-precision diamond turning has been commonly applied in the machining of cemented carbide as well. Bulla et al. [21] analyzed the effects of machining parameters and tool geometries during ductile turning of cemented carbide. Nath et al. [22] investigated ultrasonic vibration turning of cemented carbide with a polycrystalline diamond (PCD) tool and found it results in better machining performances compared to conventional turning.

Micromilling of cemented carbide also attracts more and more attention due to its great potential in industrial applications [23]. Suzuki et al. [24] developed the PCD tool with twenty cutting edges to machine the aspheric lens mold made of cemented carbide. Cheng et al. [25] designed the PCD tool with a straight edge and then performed evaluative milling experiments on cemented carbide. Nakamoto et al. [26] studied the surface quality of micromilled cemented carbide utilizing the PCD tool with a small diameter of 0.2 mm. Zhan et al. [27] investigated the influences of machining parameters on the surface quality during micromilling of cemented carbide. Compared to the ductile turning of cemented carbide, the micromilling operation is more complex due to its small scale. However, the mentioned studies are still at the exploratory stage or evaluation of diamond tool performances. The machining mechanisms in micromilling of cemented carbide are rare at present. 

After the manufacturing problem of the diamond micro end mill has been generally solved, as in the literature mentioned above, machining mechanism studies such as multiphase material removal, tool geometries optimization, and tool wear mechanism become more and more important [28,29]. The motivation of this paper is to conduct a systematical experimental investigation on the micromilling of cemented carbide with a PCD micro end tool. The cutting force characteristics, surface formation, and tool wear mechanisms were investigated in detail. It is desirable to further improve the micromilling process of cemented carbide mold to promote its industrial applications.

## 2. Experimental Procedure

**Figure 1:**
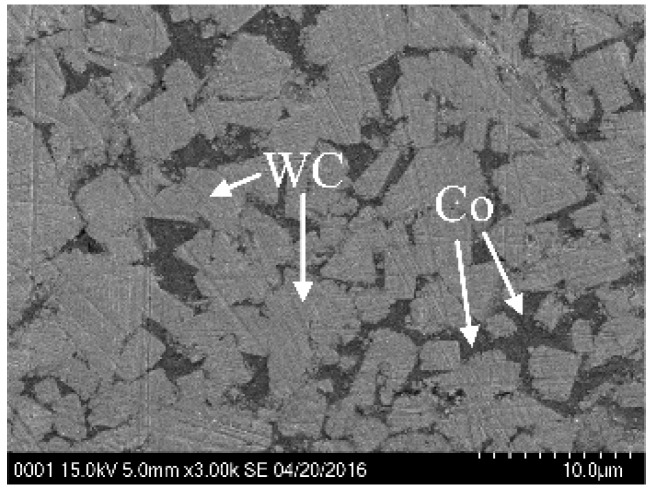
Cemented carbide workpiece.

The commercial cemented carbide WC-15Co was adopted as the workpiece material. With superior mechanical properties of high strength, good toughness, and sufficient wear resistance, it is widely used as stamping molds and extrusion molds in mass production. The large cutting force, excessive tool wear, and bad surface quality are the main problems that are faced in the micromilling process. The micrograph of used cemented carbide is shown in Figure 1. The average size of WC grains was about 4–6 μm. The cemented carbide material properties are listed in Table 1.

**Figure 2:**
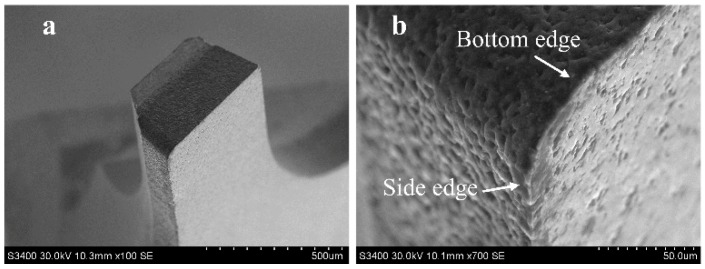
Polycrystalline diamond (PCD) micro end mill. (**a**) Overview of the tool geometry; (**b**) enlarged view of the tool tip.

A single flute PCD micro end mill that is prepared by wire electrical discharge machining (WEDM) was employed for the experiments, as shown in Figure 2. The tool diameter was 0.7 mm. It is expected to reduce the tool flection with the small ratio of length to diameter during micromilling. Therefore, the tool length was designed to be 0.5 mm. The tool tip radius was about 20 μm. The tool’s geometric accuracy of the same batch is ±1 μm. The bottom cutting edge presented an inclination angle of 10°, rake angle of 0°, and flank angle of 10°. The cutting edge radius was measured using Leica DVM500 microscope (Leica Microsystems, Buffalo Grove, IL, USA) to about 3 μm.

**Figure 3:**
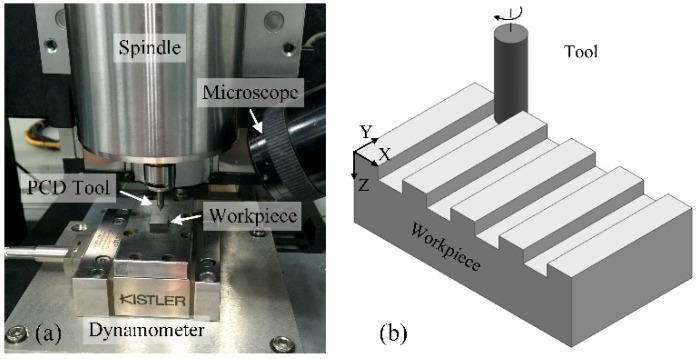
Micromilling of cemented carbide. (**a**) Experimental set-up; (**b**) schematic diagram of groove milling.

The experiments were conducted on a micromilling machine, as shown in Figure 3a. The machine can provide a positional accuracy of ±1 μm. The air-bearing spindle can rotate up to 100,000 rpm, and its radial runout is less than 1 μm. The workpiece that has been pre-polished to ensure the smooth initial surface was held on dynamometer Kistler 9256C1 to record the cutting force. The sampling rate was set at 20 kHz. Before micro milling, a fine dial test indicator was employed to guarantee the workpiece’s flatness was less than 1 μm. The stereo microscope was employed for process monitoring and tool setting. The direct micromilling experiments were performed under the condition of dry cutting to avoid the pollution of cutting coolant [30]. 

The different milling depth and feed per tooth levels were selected to contain both the ductile and brittle removal of cemented carbide during micromilling, as listed in Table 2. The spindle rotating speed was fixed at 20,000 rpm, and the corresponding cutting speed *v* was 44 m/min. Two fresh PCD micro end mills were used. First, a set of grooves were milled with the listed machining parameters to study their effect. Then, another PCD tool was used for continuous grooves milling with fixed parameters to study the tool wear, as shown in Figure 3b. The machined surface was cleaned ultrasonically in water, and then the surface roughness was measured in the middle of milled grooves along the feed direction. All the measurements were repeated four times and then averaged to obtain the results. Both the machined surface morphology and tool wear morphology were observed utilizing a scanning electron microscope. 

## 3. Results and Discussion 

### 3.1. Cutting Force Analysis 

**Figure 4:**
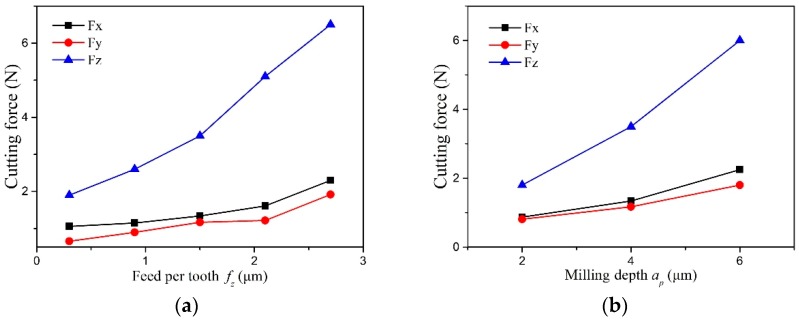
The effect of machining parameters on cutting force. (**a**) Cutting force versus feed rate (*a_p_* = 4 μm). (**b**) Cutting force versus milling depth (*f_z_* = 1.5 μm).

The cutting force signal contains fundamental information regarding the cutting process [31]. Figure 4 shows the cutting force varying with the feed per tooth and milling depth. In the three cutting force components, the axial force *Fz* is the maximum, the normal force *Fx* is the second, and the feed force *Fy* is the minimum. In micromilling, the milling depth is very small and far less than the tool tip radius. Only a small segment of the cutting edge at the tool tip bottom participates in cutting the workpiece material. This is similar to how the tool cutting edge angle is very small in the orthogonal cutting process, and it causes a very large component in the axial direction. Therefore, the recorded axial force is far greater than the other two cutting force components. In addition, the feed per tooth is also less than the cutting edge radius, and there is severe plowing under the cutting edge arc during micromilling. It may also cause the larger cutting force components in the axial direction.

With the increase of machining parameters, the three cutting force components grow in different degrees. The axial force rapidly grows from 2 N to more than 6 N. However, the growth of the normal force and feed force is relatively slight. Their changes are lower than 2 N. During micromilling, there is serious friction between the tool bottom surface and workpiece. The friction will rapidly increase with the lager machining parameters and cause a greater increase in the axial force.

### 3.2. Surface Formation Mechanism

As a hard and brittle material, cemented carbide is commonly removed with a brittle fracture in conventional cutting. It usually generates brittle damages such as micro-pits and cracks on the surface morphology. However, if the uncut chip thickness is small enough, cemented carbide can be removed with ductile cutting and obtain a surface morphology without brittle damages as well. The critical uncut chip thickness *d_c_* can be estimated using the empirical formula [32]:

(1) $$d_{c}=0.15\left(\frac{E}{H}\right){\left(\frac{K_{IC}}{H}\right)}^{2}$$

where *E* is elastic modulus, *H* is material hardness, and *K_IC_* is fracture toughness. Thus, the critical uncut chip thickness for ductile cutting is calculated to be about 1.49 μm utilizing the material properties in Table 1.

**Figure 5:**
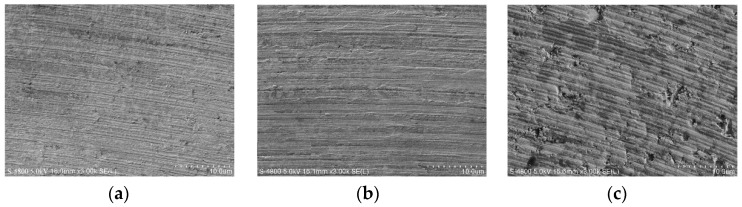
The machined surface morphology (*a_p_* = 4 μm). (**a**) *f_z_* = 0.3 μm; (**b**) *f_z_* = 1.5 μm; (**c**) *f_z_* = 2.7 μm

Figure 5 shows the surface morphology with different feed per tooth. When *f_z_* = 0.3 μm, the surface morphology characteristic exhibits regular and smooth cutting marks without any brittle damages. The surface textures confirm the material removal mode is plastic deformation, just like in the case of the machining of plastic metal. Some scale-stab can be observed on the surface morphology as well. Due to the fact that the feed per tooth is much lower than the cutting edge radius, the effective rake angle is actually large negative value. The severe plowing becomes important during the milling process. The cutting force signal also reflects the notable plowing phenomenon. Some plastic deformed material is bonded and accumulated under the cutting edge arc due to the plowing. When the accumulated material falls off, some of them will then adhere to the machined surface and form a micro scale-stab. Moreover, the small feed per tooth is comparable to the minimum uncut thickness. Some material that failed to form chips due to the minimum uncut thickness phenomenon also form a micro scale-stab on the machined surface [33]. 

**Figure 6:**
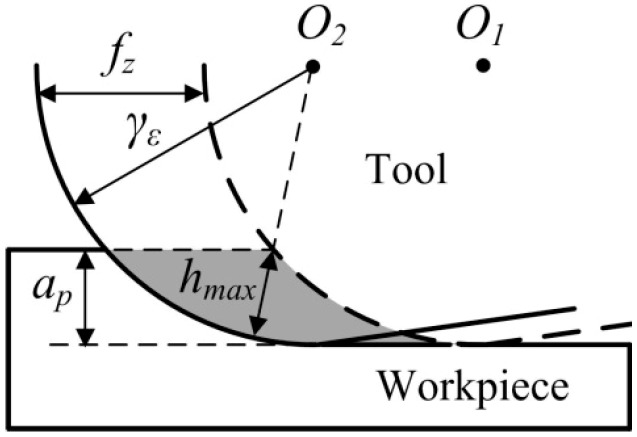
The maximum uncut chip thickness in micromilling.

It has been calculated that the critical uncut chip thickness *d_c_* for ductile cutting of cemented carbide is 1.49 μm. Although the feed per tooth *f_z_* increases to 1.5 μm, there are still no brittle damages on the surface morphology, as shown in Figure 5b. Because the tool tip radius *γ_ε_* is larger than milling depth *a_p_*, the actual uncut chip thickness is less than the nominal feed per tooth *f_z_*, as shown in Figure 6. Therefore, cemented carbide is still removed with plastic deformation. It seems that the PCD tool with large tool tip radius *γ_ε_* is conducive to perform ductile cutting of hard and brittle material. In addition, there are some side-flow burrs apparent on the surface morphology. These micro burrs are attributed to material that was subjected to high cutting stress flowing to the side of the feed mark. 

The instantaneous uncut chip thickness gradually grows from the bottom of the tool tip arc and reaches the maximum at the intersection point of the cutting edge and material surface. Through geometric calculation, the maximum uncut chip thickness *h_max_* is derived with the formula listed below:

(2) $$h_{max}=\gamma_{\varepsilon }-\sqrt{\gamma_{\varepsilon }{}^{2}+f_{z}{}^{2}-2f_{z}\sqrt{2\gamma_{\varepsilon }a_{p}-a_{p}{}^{2}}}$$

**Figure 7:**
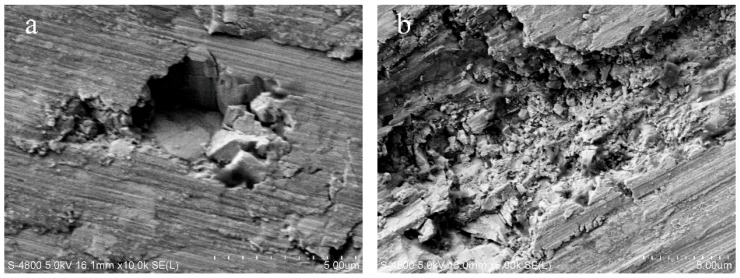
The brittle damages on surface morphology. (**a**) Micro-pit; (**b**) large pit.

From the above formula, when *f_z_* = 2.7 μm, the maximum uncut chip thickness *h_max_* reaches 1.5 μm and exceeds the critical uncut chip thickness *d_c_*. Thus, the brittle damages are exhibited on the surface morphology and greatly deteriorated the surface quality, as shown in Figure 5c. The typical brittle damages on the surface morphology are shown in Figure 7. When brittle fracture occurs, the WC particle is broken into several pieces, forming brittle cracks in itself. The broken pieces that are exposed on the machined surface are loose because they are not held by the binding phase. Then, these broken pieces fall off and the micro-pit is formed on the surface morphology, as shown in Figure 7a. The broken pieces under the machined surface are held by the binding phase and remained in micro-pit. The brittle cracks and broken pieces are still visible on the bottom of the micro-pit. Figure 7b shows that the whole WC particle is severely broken into very fine fragments. Almost all the fine fragments are dropping out and the larger pit is generated. The micro-pits and cracks greatly affect the surface quality and usually cause great surface roughness. 

**Figure 8:**
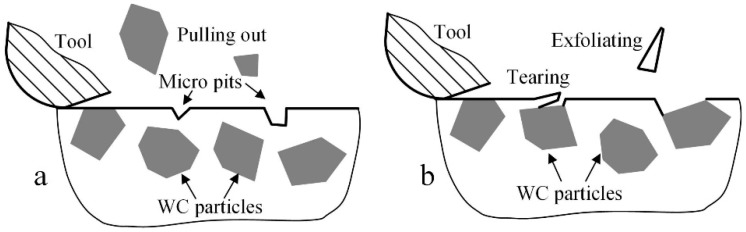
The surface damages mechanism during micromilling of the multiphase material. (**a**) Pulling out; (**b**) tearing and exfoliating.

As a multiphase material, there are also some non-cutting behaviors that affect the surface quality. As shown in Figure 8a, if there is only a small segment of WC particle left on the machined surface during the tool cutting process, it may rotate and be pulled out from the surface due to the insufficient binding strength. The pulling of the WC particle can cause a micro-pit on the surface morphology. As shown in Figure 8b, if the binding phase becomes very thin during the tool cutting process, the binding phase may tear and then exfoliate due to the severe plowing. The tearing and exfoliating behaviors can cause micro burrs or pits on the surface morphology. This surface damage mechanism may occur both in the ductile and brittle cutting. 

**Figure 9:**
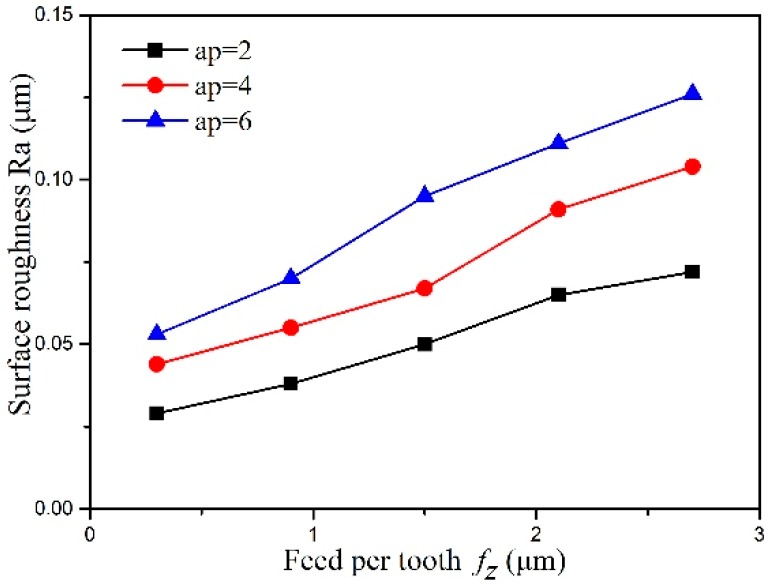
The effect of machining parameters on surface roughness.

Figure 9 shows the surface roughness gradually growing with the machining parameters. It is revealed that surface roughness growth is relatively lower since cemented carbide is removed with plastic deformation utilizing the small machining parameters. When the milling depth is 2 μm, surface roughness grows from 0.03 μm to 0.07 μm with increasing feed per tooth from 0.3 μm to 2.7 μm. The surface roughness growth is just 0.04 μm. Since cemented carbide is removed with brittle fracture utilizing the large machining parameters and surface roughness growth becomes higher relatively. When the milling depth is 6 μm, surface roughness grows from 0.05 μm to 0.13 μm with increasing feed per tooth from 0.3 μm to 2.7 μm. The surface roughness growth reaches 0.07 μm. It is indicated that surface roughness is more greatly affected by the machining parameters when the material is removed with brittle fracture rather than plastic deformation. The surface morphology mainly consisted of feed marks during ductile cutting. However, the brittle pits and cracks become significant during brittle cutting. They may rapidly propagate when increasing the machining parameters and then severely deteriorate the surface quality. This can explain the larger surface roughness growth. 

### 3.3. Tool Wear Mechanism 

**Figure 10:**
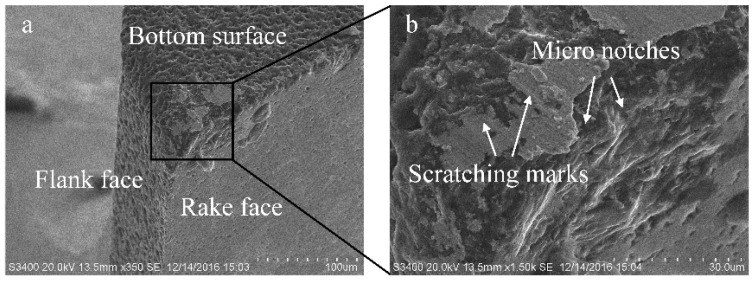
The PCD tool wear morphology. (**a**) Overview of the worn tool tip; (**b**) enlarged view of the tool wear region.

The tool wear experiments were performed with the fixed parameters of *a_p_* = 4 μm and *f_z_* = 1.5 μm. The tool tip morphology after removing cemented carbide of 3.2 mm^3^ volume is shown in Figure 10. It is seen that the PCD tool suffers serious tool wear due to the high hardness of cemented carbide. Because only a small segment of the bottom cutting edge makes contact with the workpiece, the tool wear region mainly occurred on the bottom cutting edge in the vicinity of the tool tip, including the rake face and bottom surface. There is a large piece of PCD material loss, forming a small concave on the rake face. The bottom surface is worn and becomes a rough plane. The electrical erosion pits which are left in the WEDM process are even worn flat and disappear. The cutting edge is bumpy and blunt, and the cutting edge radius has largely increased. However, the tool tip radius actually reduces due to tool wear at the bottom cutting edge. This serious tool wear usually causes an unsatisfactory machining quality. The PCD tool is invalid and in need of repair.

The tool wear mechanisms of the PCD tool mainly include microchipping, exfoliation, abrasive wear, and adhesive wear. On the bottom cutting edge, micro notches that are caused by microchipping are visible. Actually, there are only several- or even single-layer diamond particles on the cutting edge due to its sharp characteristic. Because the milling process is interrupted with the spindle rotating, the cutting edge undertakes a high-frequency mechanical crash upon the process of the tool going in and out. The periodic mechanical crash easily induces the diamond particles on the cutting edge to break inside it or peel off along its bonding surface. Subsequently, microchipping occurs, and it causes a small amount of PCD material to fall from the tool body and leads to the bumpy cutting edge, as shown in Figure 10b. 

The wearing concave is the main wear characteristic on the rake face. However, there are no obvious scratching marks or adhesions on the rake face. It is indicated that the abrasive or adhesive wear are not the main wear mechanisms on the rake face. The exfoliation is assumed to be the possible wear mechanism on the rake face. In micromilling of cemented carbide, the chips are powder sharp and include hard WC fragments. When these hard chips flow out along the rake face, they seriously damage the rake face at the same time. This behavior is sustained throughout the whole machining process and results in a serious mechanical crash on the rake face as well. Some microelectrical erosion defects that were generated in the WEDM process exist on the rake face. In addition, microchipping on the cutting edge may also induce some microcracks that propagate on the rake face. Under the serious mechanical crash, these microdefects gradually develop into material exfoliation on the rake face. The exfoliating behavior causes a large piece of PCD material to drop from the tool body. Because the serious mechanical crash that was induced by the hard chips is only focused on the bottom of the rake face, the exfoliating pits are mainly formed on the bottom of rake face as well, as shown in Figure 10b. 

**Figure 11:**
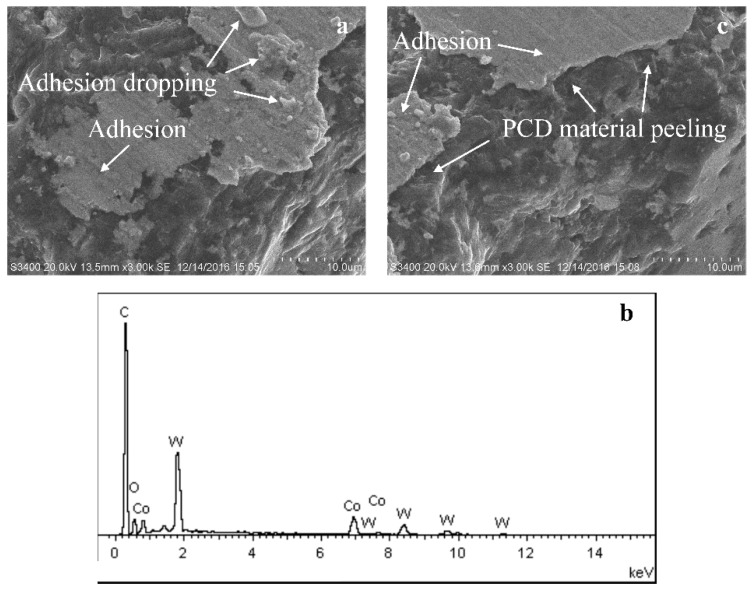
The adhesive wear mechanism on bottom surface. (**a**) Adhesions on bottom surface; (**b**) element compositions of adhesions; (**c**) dropping of tool material.

The scratching marks are clear to see on the bottom face. This indicates the severe abrasive wear during the micromilling process due to the high hardness of cemented carbide. Moreover, there are many visible adhesions on the bottom surface, as shown in Figure 11a. It is just like the state of the machining process of plastic metal. These adhesions are identified to be cemented carbide material which is in a serious plastic deformation state, based on the energy dispersive spectrometer (EDS) results in Figure 11b. This illustrates that the hard and brittle material is removed with ductile cutting during micromilling as well. From the cutting force results, it can be seen that the axial force component is very large. Because the machining parameters are only several microns, the axial force load is concentrated on the cutting region of only a few square microns. This generates very high cutting stress on the bottom surface. Cemented carbide material is adhered and embedded into the tool surface under the high cutting stress. With the accumulation of adhesive material, the adhesive layer becomes the bulgy part on the tool surface. The bulgy adhesive layer suffers severe friction and a high-frequency of attacks. This leads to some adhesions fracturing and falling from the tool body. Meanwhile, some diamond particles also peel from the tool surface and form a rough region on the tool surface, as shown in Figure 11c. Then, the new adhesive layer will generate and the adhesive wear process will occur again. The severe adhesive wear is the dominant wear mechanism on the bottom surface.

**Figure 12:**
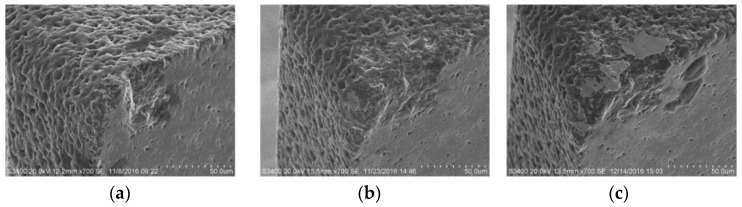
The effect of material removal volume on tool wear morphology. (**a**) *V* = 0.4 mm^3^; (**b**) *V* = 1.6 mm^3^; (**c**) *V* = 3.2 mm^3^.

With a continuous milling process, more and more material has been removed, and the tool wear is gradually increasing as well. Figure 12 shows the PCD tool wear process varying with material removal volume *V*. Although only a 0.4 mm^3^ volume of cemented carbide is removed, a large wear area appeared on the rake face; however, there is just slight wear on the bottom surface. In the early stage, due to the electrical erosion defects on the tool surface, the tool wear behaviors are mainly microchipping on the cutting edge and exfoliating on the rake face. After the electrical erosion pits have disappeared, these two kinds of tool wear behaviors are reduced and become minor. With the increase of the material removal volume to 1.6 mm^3^, the wear area on the rake face has increased little. However, the bottom surface wear is obvious due to its large increase, as shown in Figure 12b. The severe abrasive and adhesive wear behaviors are dominant at this stage with the increasing contact between the bottom surface and workpiece. When the material removal volume reaches 3.2 mm^3^, tool wear is very serious both on the rake face and bottom surface, as shown in Figure 12c. The extrusion and friction between the worn tool and workpiece are rising rapidly. This results in the adhesive wear process becoming more notable. 

**Figure 13:**
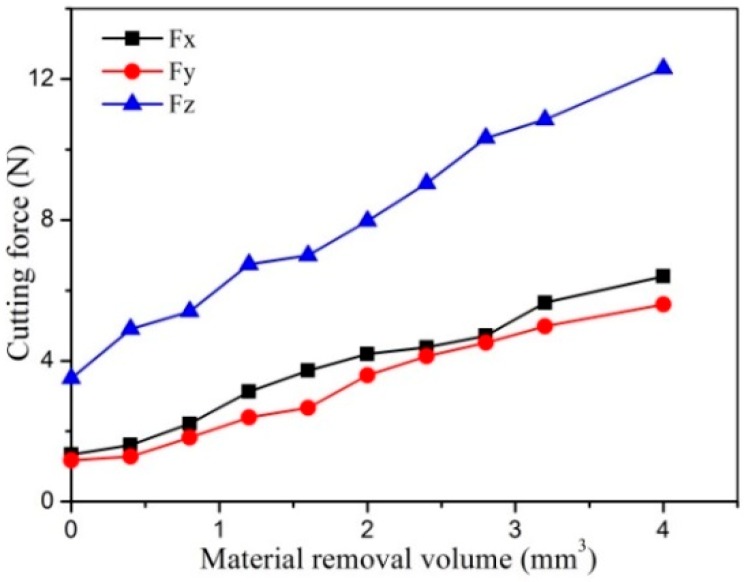
The effect of material removal volume on cutting force.

The cutting force variation as a function of the material removal volume is shown in Figure 13. It is seen that the cutting force almost linearly grows with the material removal volume. Respectively, the normal force *Fx*, feed force *Fy*, and axial force *Fz* grows from 1.3 N, 1.2 N, and 3.5 N to 6.4 N, 5.6 N, and 12.3 N, respectively, after removing cemented carbide with a volume of 4 mm^3^. They have increased almost four times due to severe tool wear. The large cutting force can greatly increase the flection deformation and breakage risk of the PCD tool due to its small diameter and low strength.

**Figure 14:**
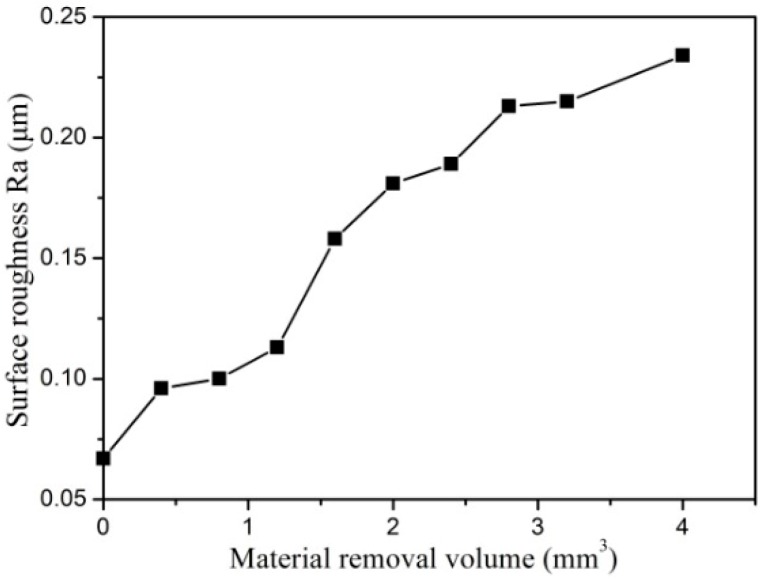
The effect of material removal volume on surface roughness.

Figure 14 shows that the surface roughness increases with the material removal volume as well. When the PCD tool is fresh and sharp, the surface roughness is very low, at about 0.07 μm. After 4 mm^3^ of cemented carbide was removed, the PCD tool was sufficiently worn to become blunt, and the surface roughness increased to 0.24 μm. The surface quality severely deteriorated with the tool wear. This indicates that the PCD tool is invalid and unsuitable for continuous machining. 

## 4. Summary and Conclusions 

This paper presents an experimental investigation on the direct micromilling of cemented carbide with a PCD tool that was prepared by WEDM. Based on the results, the following conclusions can be drawn:

- The PCD tool with a large tool tip radius is conducive to perform ductile cutting of cemented carbide. The damage features on the ductile cutting surface are micro scale-stab and burrs that are attributed to the severe plowing and material side-flow. Micro-pits and cracks are the main damage features on the brittle cutting surface morphology. The machining parameters present a greater influence on the surface roughness when material is removed with brittle fracture compared to plastic deformation due to the different surface morphology characteristics.
- The PCD tool wear region is focused on the bottom cutting edge near the tool tip. The tool wear mechanisms mainly include microchipping on the cutting edge, exfoliation on the rake face, and abrasive and adhesive wear on the bottom face. The tool wear process shows microchipping and exfoliating in the early stage, and subsequently, severe abrasive and adhesive wear in the later stage.

## Figures and Tables

**Table 1:** Cemented carbide properties.

Properties.	Density (g/cm^3^)	Hardness (HV_10_)	Elastic Modulus (GPa)	Fracture Toughness (MPa·m^1/2^)
**Value**	13.9	1607	498	9.1

**Table 2:** Micromilling parameters.

Parameters	Cutting Speed *v* (m/min)	Milling Depth *a_p_* (μm)	Feed per Tooth *f_z_* (μm)
**Value**	44	2, 4, 6	0.3, 0.9, 1.5, 2.1, 2.7
